# The latent HIV reservoir: current advances in genetic sequencing approaches

**DOI:** 10.1128/mbio.01344-23

**Published:** 2023-10-09

**Authors:** Preeti Moar, Thomas A. Premeaux, Andrew Atkins, Lishomwa C. Ndhlovu

**Affiliations:** 1 Department of Medicine, Division of Infectious Diseases, Weill Cornell Medicine, New York City, New York, USA; 2 Feil Family Brain and Mind Research Institute, Weill Cornell Medicine, New York City, New York, USA; Albert Einstein College of Medicine, Bronx, New York, USA

**Keywords:** HIV persistence, latency, DNA, RNA, virus, antiretroviral therapy, remission, cure

## Abstract

Multiple cellular HIV reservoirs in diverse anatomical sites can undergo clonal expansion and persist for years despite suppressive antiretroviral therapy, posing a major barrier toward an HIV cure. Commonly adopted assays to assess HIV reservoir size mainly consist of PCR-based measures of cell-associated total proviral DNA, intact proviruses and transcriptionally competent provirus (viral RNA), flow cytometry and microscopy-based methods to measure translationally competent provirus (viral protein), and quantitative viral outgrowth assay, the gold standard to measure replication-competent provirus; yet no assay alone can provide a comprehensive view of the total HIV reservoir or its dynamics. Furthermore, the detection of extant provirus by these measures does not preclude defects affecting replication competence. An accurate measure of the latent reservoir is essential for evaluating the efficacy of HIV cure strategies. Recent approaches have been developed, which generate proviral sequence data to create a more detailed profile of the latent reservoir. These sequencing approaches are valuable tools to understand the complex multicellular processes in a diverse range of tissues and cell types and have provided insights into the mechanisms of HIV establishment and persistence. These advancements over previous sequencing methods have allowed multiplexing and new assays have emerged, which can document transcriptional activity, chromosome accessibility, and in-depth cellular phenotypes harboring latent HIV, enabling the characterization of rare infected cells across restrictive sites such as the brain. In this manuscript, we provide a review of HIV sequencing-based assays adopted to address challenges in quantifying and characterizing the latent HIV reservoir.

## INTRODUCTION

An estimated 38 million people in the world are living with HIV-1 (HIV) and the advent of effective antiretroviral drug therapy (ART) has significantly improved survival in individuals with HIV who have access. However, strict continuous adherence remains a key to prevent viral recrudescence from reservoir sites (1). HIV enters and integrates into the host genome of resting memory CD4+ T cells and other cell types (2) in the peripheral blood, lymphoid organs, gut mucosa, and the central nervous system (CNS), establishing reservoirs early during the acute stages of infection. Early ART initiation can limit the size of an established reservoir and the diversity of the replication-competent provirus (3). However, cells that harbor latent HIV can undergo clonal expansion and persist for years despite ART (4, 5). These stable and dynamic HIV reservoirs in multiple cellular and anatomical tissue sites remain a major barrier to HIV cure (6). Various approaches are being pursued to eradicate HIV, predominately stem cell transplantation (7 – 9), shock and kill (10, 11), block and lock (12, 13), gene editing (14 – 16), and immunotherapies (17 – 23). With the exception of a handful of cases through either stem cell transplantation to treat life-threatening cancers (24 – 28) or natural control (29, 30), a successful cure remains a formidable challenge in people with HIV. Thus, an accurate understanding of the HIV reservoir and its dynamics will be key to developing effective strategies to eliminate or permanently silence HIV (31, 32).

The small number of cells harboring latent HIV during suppressive ART has been a focus of HIV research over the years (33, 34). A thorough understanding of reservoir features, quality, and state is necessary to pursue an effective remission strategy and achieve HIV eradication in the absence of ART. Although CD4+ T-cell subsets are regarded as the predominant cellular compartment of the HIV reservoir, monocytes and macrophages have emerged as important contributors to HIV persistence (35 – 37). Tissue macrophages in seminal vesicles (38 – 40), the urethra (41, 42), adipose tissue (43, 44), and liver tissue (45, 46), as well as follicular dendritic cells (47) and hematopoietic progenitor cells (48) have all been shown to harbor HIV provirus during ART. HIV provirus has also been detected in the lungs (49) and CNS-resident cells, particularly microglia (50) and astrocytes (51, 52). Moreover, HIV latency is regulated at multiple molecular levels in transcriptional and post-transcriptional processes. Additionally, most proviruses contain large internal deletions, the majority of proviral sequences are replication-defective and there is significant heterogeneity in the sequence composition, genomic location, stability, and expression of the HIV reservoir both within and between individuals. In the pursuit of accurate methods to measure and characterize the HIV reservoir, rigorous efforts have been made in recent years with significant success.

Advances in sequencing approaches have enabled researchers to improve the resolution of the latent HIV reservoir in unprecedented detail. The advent of single-cell sequencing (53) allows for the characterization of individual cells in complex populations and has played an important role in exploring the heterogeneity of the HIV reservoir (54, 55). Integration site analysis by sequencing has allowed for the identification of the locations in the host genome where HIV has integrated (56), while T-cell receptor (TCR) sequencing (57) informs about the clonal expansion of the provirus. Long-read sequencing technologies can produce reads that span the entire HIV genome, enabling the detection of rare and complex variants (58, 59). These sequencing approaches have advanced our understanding of the latent HIV reservoir and have provided insights into the mechanisms of HIV persistence. In this review, we discuss techniques to measure the HIV reservoir, with a specific focus on new methods employing state-of-the-art DNA sequencing technologies that are constantly evolving.

### Non-sequencing-based methods to measure and characterize the HIV reservoir

HIV curative studies commonly measure levels of total proviral DNA, intact provirus, transcriptionally competent provirus (viral RNA), translationally competent provirus (viral protein), and replication-competent virus to assess HIV reservoir size and dynamics. These rely on either directly measuring the proviral genomes by PCR-based assays or by measuring the basal, experimentally inducible, persistent HIV using viral outgrowth assays. Only replication-competent proviruses are sources of viral rebound after ART cessation. The quantitative viral outgrowth assay (QVOA) has been the gold standard to measure replication-competent HIV and provides a definitive minimum estimate of reservoir size. Purified resting CD4+ T cells plated in a limiting dilution format are stimulated with a T-cell activator to induce viral production (60). The exponential expansion of released virus by propagation in cells susceptible to infection allows the detection of viral antigen from a single latently infected cell. However, the high cost, labor requirements, and high number of cells required for QVOA limit its application.

Modifications of the QVOA have improved the assay throughput and have included more efficient induction of the virus by the use of antibody-mediated TCR stimulation (61, 62) or protein kinase C activation (63) as opposed to phytohemagglutinin (PHA) (64). CD8-depleted peripheral blood mononuclear cells (PBMCs) obtained from uninfected donors have traditionally been used to promote viral outgrowth; however, the assay can also be applied to bulk PBMCs (65), differentiated CD4+ T cells (66), or cell lines that support HIV replication (67, 68). QVOA has also been used to measure functional latent reservoirs in CD4+ T cells and macrophages within the spleen, lung, and brain of simian immunodeficiency virus (SIV)-infected macaques (69, 70). The Macrophage QVOA, also named MΦ-QVOA, enables the assessment of blood monocytes and tissue-resident macrophages that may also harbor replication-competent proviruses (71, 72). Furthermore, the murine QVOA (mVOA) was developed to monitor replication-competent provirus *in vivo* by adoptive transfer of CD4+ T cells derived from donors with HIV on suppressive ART to NOD/scid gamma immunodeficient mice (73, 74). An alternative approach to mVOA is the humanized mice QVOA, which provides a more relevant physiological setting and was found to be more sensitive than standard QVOA (75).

While the QVOA provides definitive evidence of the persistence of latent HIV, it does not capture all replication-competent proviruses with a single round of stimulation and additional viral outgrowth can be induced with multiple rounds of T-cell activation (5, 76). Additionally, our knowledge about HIV reactivating agents and the nature of the viral reservoirs that respond to them is limited. Further study of mechanisms of HIV reactivation and identification of novel latency-reversing agents (LRA) that can reactivate HIV are essential to characterize the viral reservoir pool accurately. However, these modified multiple-stimulation QVOAs are not experimentally feasible for cure trials; therefore, the QVOA is considered a definitive minimum estimate but not a conclusive measure for the size of the latent reservoir.

PCR-based methods such as quantitative real-time PCR and digital droplet PCR (ddPCR) have been widely used to estimate the frequency of HIV reservoirs (77, 78). As the vast majority of HIV proviruses are defective, these estimates do not provide information regarding intactness or inducibility. However, assays such as intact proviral DNA assay (IPDA) provide more qualitative data as compared to quantitative PCR assays (79). Proviral amplification at both Ψ and env regions via multiplex ddPCR enables the detection and classification of intact and hypermutated proviruses (80). Expanded IPDA approaches can include global HIV subtypes (81) or rather quantify proviral RNA (intact viral RNA assay) (82). The individual proviral sequencing assay, an integrated two-step assay, can identify the proviral integration sites, intact sequences, and their competent/defective status (83). Q4PCR, and similar assays, which combine quadruplex quantitative PCR and next-generation sequencing (NGS) can additionally assess genetic diversity to better understand clonal dynamics over time (84). Reservoir decay measurements by Q4PCR are quantitatively similar to viral outgrowth assay and IPDA with the addition of sequence information that distinguishes intact and defective proviruses.

Proviruses that are not fully replication-competent can still be capable of transcribing viral mRNAs and translating viral proteins (85 – 87). Rare subsets of infected cells have been identified, which express HIV mRNA and proteins without spreading infection (88). These cells can cycle back to a latent state and contribute to the latent HIV reservoir. Viral replication during ART can be measured using ultrasensitive single-copy HIV RNA assays to evaluate inducible persistent HIV, which can have a detection rate of 1 HIV RNA copy/mL of plasma (89). Single-cell-in-droplet PCR is used to estimate the frequency of RNA-expressing cells, allowing the isolation and enumeration of individual latently infected lymphocytes and other tissue-derived cells (90). Other RNA PCR approaches use limiting dilution to estimate the frequency of HIV cell-associated RNA-expressing cells, such as the Tat-/rev-induced limiting dilution assay (91, 92). This approach reduces reservoir frequency overestimation and can be expanded to detect all major HIV genetic subtypes with comparable sensitivity (93). Initially, *in situ* hybridization (ISH) was used to detect HIV mRNA in the lymph nodes of people with HIV during viral suppression (94). Next-generation ISH assays, such as RNAscope and fluorescence *in situ* hybridization (FISH)-flow, enable the detection of single HIV RNA-positive cells via microscopy (95) or flow cytometry (96, 97) and allow high-throughput and high-sensitivity detection while reducing off-target binding of the probes. These approaches also preserve tissue integrity and allow spatial interrogation of the microenvironment (98, 99), which is important in the context of tissue-resident HIV reservoirs such as in the brain.

While not all cells harboring HIV mRNA or viral proteins contain replication-competent proviruses, the elimination of transcription- and translation-competent proviruses should be considered in the context of cure. Quantification of baseline or inducible expression of viral proteins (i.e., Gag) demonstrates that translationally competent virus can be detected at a frequency of one copy per million peripheral blood mononuclear cells in virally suppressed persons with HIV but has a high false discovery rate. Several methods have been implemented to overcome this limitation, including combining fiber-optic array scanning technology and automated digital microscopy to detect gag-positive cells with concomitant CD4 downregulation (100), the use of two distinct gag protein probes in flow cytometry (101), or using Env-specific bNAbs combined with magnetic-enrichment and fluorescence-assisted cell-sorting to enrich activated latently infected cells (latent cell capture, or LURE) (54).

While these assays provide a fair quantification of the HIV provirus, the associated high cost, labor intensiveness, and biosafety requirements limit their application in cure-directed studies. QVOA and its derivatives have been the gold standard for measuring replication-competent HIV but greatly underestimate the size of the reservoir, as it requires amplification of the virus by external stimulations that not all reservoirs may respond to. On the other hand, IPDA offers a promising strategy to estimate the size of intact HIV populations in a more cost-effective and labor-effective manner but overestimates the size of the reservoir as it lacks the ability to disregard all defective proviruses. The median frequency of cells carrying intact proviruses detected by IPDA is more than 60-fold higher than what is detected by the standard QVOA (60). These caveats of the PCR-based assays are easily overcome by the emerging sequencing methods by providing the essential missing information, i.e., the genetic identity of the provirus. Single-genome, single-cell, full-length, and long-read sequencing technologies are valuable tools for the understanding of complex multicellular processes in a diverse range of tissues and have successfully been employed to measure and characterize the HIV reservoir.

### Sequencing methods to measure and characterize the HIV reservoir

**Fig 1:**
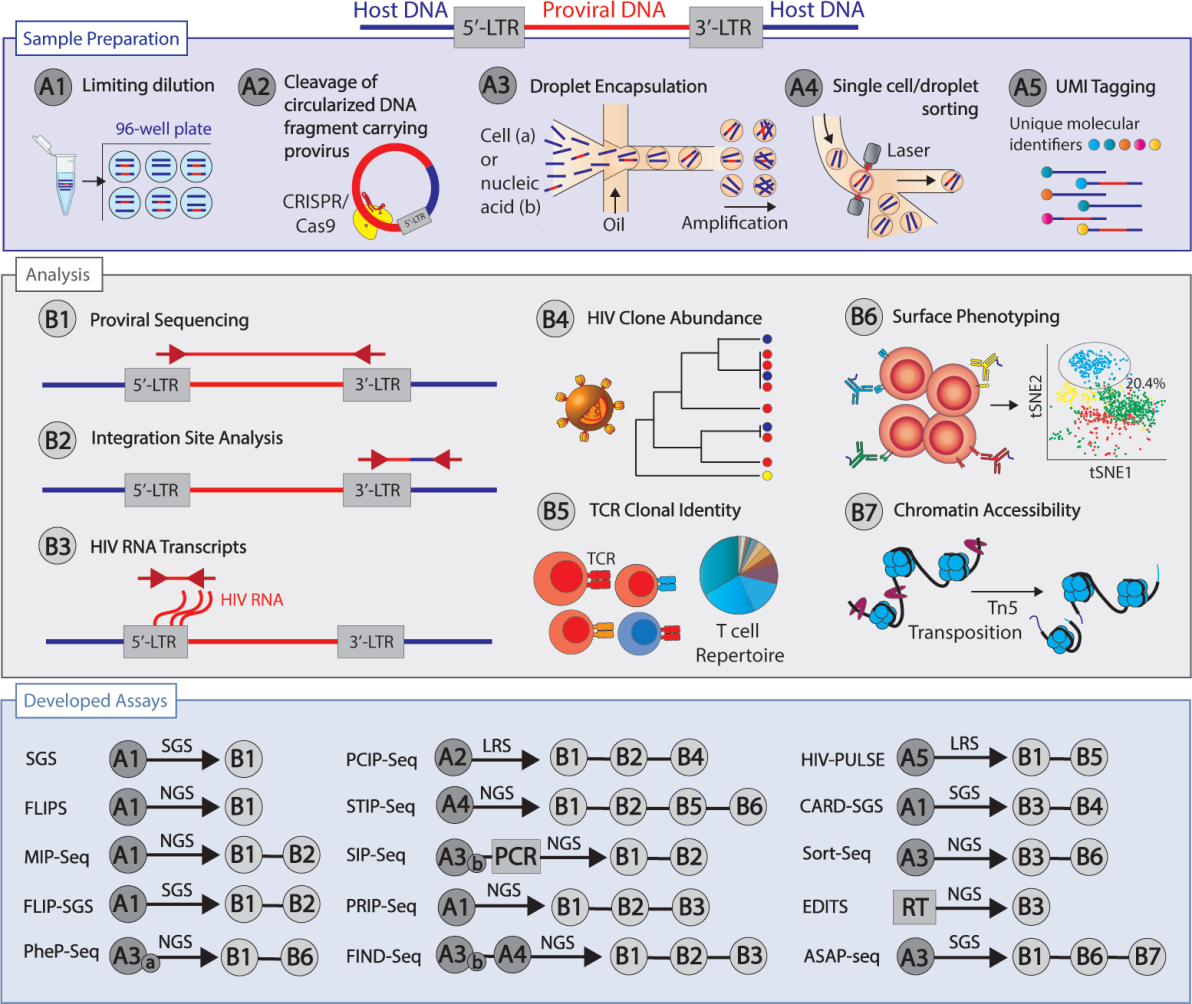
Overview of DNA and RNA sequencing methodology to characterize the HIV reservoir. Summary of the sample preparation and analyses options and specific developed assays. LRS, long read sequencing and RT, reverse transcription. For full naming of the assays, see Table 1.

The development of sequencing assays that measure HIV persistence has been invaluable for understanding the nature of the latent reservoir. Along with the genetic sequence of the provirus, these assays have the ability to inform the distribution and integration sites of HIV in the host genome. These features have been summarized in Table 1. Current advancements in the traditional sequencing methods have allowed multiplexing and development of new assays that can measure transcriptional activity, chromosome accessibility, and phenotype of the cells harboring latent HIV (Fig. 1). Here, we review the sequencing methods that are used to measure and characterize the HIV reservoir; however, the list of assays available is non-exhaustive as the field progresses with an incredible pace.

**TABLE 1:** Summary of current HIV sequencing methods available to characterize the HIV reservoir

Method	Target	Features	Cell type	Limitations	Reference
Single genome sequencing	DNA	Sub-genomic amplification followed by Sanger sequencing.	CD4+ T cells, lymph nodes, GALT, PBMCs	Low sensitivity, low throughput, and short-read sequencing.	( 102, 103, 104 – 106)
Full-length individual proviral sequencing	DNA	Two-step nested PCR at limiting dilution followed by NGS to sequence (intact and defective) single HIV proviruses.	CD4+ T cells	Intact proviruses identified by FLIPS may not be truly replication competent as it lacks integration site analysis.	(107)
Cell-associated HIV RNA and DNA single-genome sequencing	RNA	Improved isolation of intracellular RNA from PBMCs with end-point dilution analysis of HIV RNA-expressing cells by SGS.	PBMCs	Low throughput and labor-intensive,	(108)
Envelope detection by induced transcription-based sequencing	RNA	Reverse transcription of RNA from LRA-induced cells, followed by barcoding and NGS to measure inducible cell-associated HIV RNA.	CD4+ T cells	Needs external stimulation by LRA, hence underestimates the total inducible HIV reservoir.	(109)
Single-cell RNA sequencing	RNA	RNA from single cells is reverse transcribed to form cDNA, followed by NGS.	CD4+ T cells, PBMCs, CSF, monocytes	Underestimates reservoir size as it may not detect all latent provirus due to lack of active transcription.	(52, 54, 55, 110)
Matched integration site and proviral sequencing	DNA	Near-full-length HIV sequencing (NGS) and corresponding chromosomal integration site analysis.	CD4+ T cells	Low throughput and does not provide information about transcriptional activity.	(56)
Full-length integrated proviral single-genome sequencing	DNA	Near-full-length HIV sequencing (SGS) and corresponding chromosomal integration site analysis.	PBMCs and LNMCs	Low throughput and short read sequencing.	(111, 112)
Sort-Seq	RNA	Single-cell transcriptome analysis of sorted HIV + cells.	CD4+ T cells	Needs external stimulation by LRA, thus underestimates the total inducible HIV reservoir.	(113)
Single-cell near full-length sequencing	DNA	Single sorted cells are subjected to near full-length amplification using a modified FLIPS assay.	CD4+ T cells	Needs external stimulation by LRA, thus underestimates the total inducible HIV reservoir.	(114)
Pooled CRISPR inverse PCR sequencing	DNA	Long-read sequencing followed by simultaneous identification of the proviral integration site and clone abundance.	CD4+ T cells	HIV proviruses captured by PCIP-seq were only partially sequenced (~ 2.4 kb).	(58)
Simultaneous TCR, integration site and provirus sequencing	DNA	HIV-flow assay followed by near-full-length proviral sequencing, integration site analysis, TCR β sequence analysis, and phenotyping of the host cell.	CD4+ T cells	Needs external stimulation by LRA, thus underestimates the total inducible HIV reservoir.	(57)
SIP-Seq	DNA	Full-length DNA sequencing of HIV + microfluidic droplets and corresponding chromosomal integration site analysis.	CD4+ T cells	Does not provide information about host cell phenotype and transcriptional activity.	115)
Parallel HIV-1 RNA, integration site and proviral sequencing	DNA, RNA	Near-full-length HIV sequencing (NGS), corresponding integration site analysis and transcriptional activity.	CD4+ T cells	Low throughput, labor-intensive, costly.	(116)
Focused interrogation of cells by nucleic acid detection and sequencing	DNA, RNA	Cells are lysed and encapsulated in microfluidic droplets. HIV DNA + droplets are captured for transcriptome analysis.	CD4+ T cells	Not single-cell and does not differentiate between intact and defective provirus.	(117, 118)
Assay of transposon-accessible chromatin sequencing (ATAC-Seq)	DNA	Direct *in vitro* transposition of sequencing adaptors into accessible chromatin regions.	CD4+ T cells, microglia	Does not measure the size of the HIV reservoir, microglia studies limited to cell lines.	(119, 120)
ATAC with select antigen profiling by sequencing	DNA	Single-cell ATAC-Seq with simultaneous measurement of cell surface and intracellular protein markers.	CD4+ T cells, Monocytes	Short read sequencing and only detects provirus in the accessible chromatin.	(121)
PheP-Seq	DNA	Antibody-tagged cells are encapsulated in microfluidic droplets, followed by single-cell barcoding and NGS.	CD4+ T cells, lymph nodes	Does not provide information about integration sites and transcriptional activity.	(122)
HIV proviral UMI-mediated long-read sequencing	DNA	Unique molecular identifiers-tagged HIV provirus is amplified and barcoded for high throughput long-read sequencing.	CD4+ T cells	Does not provide information about host cell phenotype, integration sites, and transcriptional activity.	(123)

#### DNA sequencing

Single genome sequencing (SGS) involves the sequencing of individual copies of the proviral DNA and targets sub-genomic regions of the HIV genome using the Sanger sequencing method (102, 124, 125). SGS-based proviral sequencing assays provide important information about proviral diversity (103 – 106) but overestimate the amount of replication-competent virus, as Sanger sequencing fragments are typically in the range of 1–2 kb in length and do not capture the full-length provirus. To address this limitation, several near-full-length (NFL) sequencing assays have been developed. The initial NFL HIV assay involved the amplification of four overlapping segments of a single HIV genome, which were then sequenced and consolidated (76). This method allowed for the identification of defective versus intact HIV genomes and revealed that the latent HIV reservoir was underestimated by earlier SGS-based assays. The presence of defective proviruses capable of transcribing unspliced HIV-RNA species was found in patients at all stages of HIV infection (86). NFL sequencing has also been used to longitudinally study the proviral landscape of individuals on ART to investigate the selective pressures influencing HIV reservoir dynamics (126). The advent of NGS has allowed for in-depth high throughput genome-scale analyses of persistent HIV, overcoming the limitations of SGS.

The full-length individual proviral sequencing (FLIPS) assay uses NGS to determine the distribution of latent, replication-competent HIV within memory CD4+ T-cell subsets in individuals on long-term ART (107). Two rounds of nested PCR at limiting dilution targeting the highly conserved 5′ and 3′ long terminal repeat (LTR) regions are used to amplify approximately 9 kb of the HIV genome, followed by high-throughput NGS. The FLIPS assay does not require the proviruses to be reactivated, like in earlier assays, and uses a stringent approach to eliminate defective sequences. Another group used a similar approach to profile HIV reservoirs in unfractionated PBMCs, *ex vivo* isolated CD4+ T cells, and subsets of functionally polarized memory CD4+ T cells (127). Full-length sequencing could identify multiple sets of independent, NFL proviral sequences from ART-treated individuals that were completely identical, consistent with the concept of clonal expansion of CD4+ T cells harboring intact HIV. FLIPS can provide an accurate and comprehensive picture of viral diversity within an individual or a population of infected individuals, but it does not provide information about the genetic location of the provirus.

An important advancement to the FLIPS assay was to link the full-length HIV genome sequences to their integration site. FLIPS with SGS (FLIP-SGS) was applied to PBMCs from an ART-suppressed donor living with HIV and yielded integration sites of four genomes that appear to contain large internal deletions (128). This assay defined clonal expansion of infected CD4+ T cells as a mechanism that maintains the HIV reservoir and as the source of identical sequences observed during therapy and rebound, rather than from ongoing replication (111, 112). More recent efforts have used multiple displacement amplification (MDA) followed by NGS to invent the matched integration site and proviral sequencing (MIP-Seq) assay (56). DNA material from each individual MDA reaction was split and separately subjected to viral sequence amplification with primers spanning NFL HIV and to chromosomal integration site analysis based on integration site loop amplification (4). Early studies using this method suggest that in participants on durable therapy, intact proviruses are enriched in non-genic regions of the genome (56).

Methods such as MIP-Seq rely on whole genome amplification of isolated HIV genomes with separate reactions to identify the integration site and sequence the associated provirus. As a result, these methods are quite labor-intensive, limiting the number of proviruses one can assess. To exploit the potential of long reads, a pooled CRISPR inverse PCR sequencing (PCIP-Seq) assay was developed, which leverages selective cleavage of circularized DNA fragments carrying proviral DNA with a pool of clustered regularly interspaced short palindromic repeats (CRISPR) guide RNAs, followed by inverse long-range PCR and multiplexed sequencing on the Oxford Nanopore Technologies (ONT) platform (58). This method allows the simultaneous identification of the integration site and tracks clone abundance while also sequencing the provirus inserted at that position. While Illumina-based techniques require access to a number of capital-intensive instruments, PCIP-seq libraries can be generated, sequenced, and analyzed with the basics found in most molecular biology labs and preliminary results are available just minutes after sequencing begins, which makes this method more relevant for resource-limited clinical settings. However, the majority of HIV proviruses captured by PCIP-seq were only partially sequenced (on average approximately 2.4 kb), probably due to the low proviral load and limited clonal expansion observed in people with HIV on long-term ART (58).

Long-read sequencing, also known as third-generation sequencing, has revolutionized genomics research in the past decade with the ability to resolve some of the most challenging regions of the human genome (129, 130). While NFL sequencing assays are based on labor-intensive and costly principles of repeated PCRs at limiting dilution restricting their scalability, long-read sequencing methods can typically cover >10  kb with 87%–98% accuracy from single DNA molecules without the need of PCR amplification (131). HIV proviral UMI-mediated long-read sequencing (HIV-PULSE) is a high-throughput, long-read sequencing assay that allows for the characterization of the HIV proviral landscape (123). This assay uses unique molecular identifiers (UMIs) to tag individual HIV genomes, allowing for the omission of the limiting dilution step and enabling long-range PCR amplification of many NFL genomes in a single PCR reaction, while simultaneously overcoming poor single-read accuracy. When compared to the widely applied FLIPS assay, HIV-PULSE shows similar sensitivity and overall good concordance, at a significantly higher throughput (123). Another full-length sequencing approach was recently used to profile viral and host cell transcripts simultaneously from unamplified cDNA (132). This technique uses a novel one-step chemical ablation of 3′ RNA ends, which doubles the breadth of coverage and increases the detection of long transcripts (>4 kb). The approach was successfully used to interrogate host cell and HIV-1 transcript dynamics upon reactivation in the J-Lat latency model (132).

The simultaneous integration site and provirus sequencing (SIP-Seq) assay uses whole-genome amplification in microfluidic droplets to amplify the HIV genome in its native context, and TaqMan PCR to tag droplets containing proviruses for sequencing (115). The result is a technology providing the full-length virus genome connected to its host-cell junctions in a single continuous assembly without the need for two separate reactions. The speed and efficiency of droplet microfluidics allow recovery of single provirus genomes in a 150-million-fold higher background of DNA (133, 134). However, the assay lacks information about the transcriptional activity and phenotype of the cells harboring the detected HIV provirus. The simultaneous TCR, integration site, and provirus sequencing (STIP-Seq) assay enables sequencing of the proviral genome and matched integration site of translation-competent proviruses, as well as phenotypic characterization and TCR sequencing of the host cell (57). STIP-Seq is a derivative of the HIV-flow assay (101). DNA from p24+ cells is amplified by MDA, before performing NFL proviral genome sequencing, integration site analysis, TCR sequencing, and *post hoc* determination of the CD4+ T-cell memory phenotype. Applying STIP-Seq to PBMCs from individuals living with HIV on stably suppressive ART suggested that cell clones harboring translation-competent proviruses contribute to residual viremia and viral rebound upon ART interruption (57).

While all the assays discussed above focus on proviral DNA sequencing, recent advancements highlight the assays that can integrate whole-genome sequencing with transcriptional profiling. Parallel HIV-1 RNA, integration site and proviral sequencing (PRIP-Seq) is a multidimensional assay for HIV reservoir profiling, which was designed by a combination of the MIP-Seq assay (56) with a protocol for simultaneous genome and transcriptome analysis, called G&T Seq (135). PRIP-Seq is capable of the parallel interrogation of HIV RNA, chromosomal integration sites, and the corresponding proviral sequence of individual HIV-infected cells (116). A limitation of the study was that the transcriptional profiles of individual proviruses were related to chromatin features from reference data sets and not from single-cell assessments in infected cells. Nevertheless, it can serve as a great tool for the global mapping of transcriptionally active and silent proviruses relative to genome-wide genomic and epigenetic chromatin features and the identification of proviral species that are under evolutionary selection pressure during ART *in vivo*.

Microfluidics based focused interrogation of cells by nucleic acid detection and sequencing (FIND-Seq) can accurately detect and isolate the transcriptomes from rare HIV DNA+ cells. FIND-Seq separates millions of single cells within water-in-oil droplets for immediate lysis, followed by polyadenylated RNA sequence recovery and sorting according to HIV DNA detection (117, 136). This approach isolates whole transcriptomes from cells containing quiescent viruses without the need for *in vitro* latency reversal, thereby capturing a transcriptome-wide profile of these cells in their natural state. A major caveat of FIND-Seq is the limited ability to infer theintactness or replication competence of the provirus. However, it has the potential to investigate previously inaccessible cells, including rare cell subsets. This method was recently used to identify a therapeutically targetable mechanism that limits XBP1-driven pathogenic astrocyte responses (118). Since astrocytes are an important HIV reservoir that has not been sufficiently explored, methods like FIND-Seq are an attractive approach to expand the horizon.

The transposase-accessible chromatin using sequencing (ATAC-seq) assay is based on direct *in vitro* transposition of sequencing adaptors into native chromatin and is a rapid and sensitive method for integrative epigenomic analysis (137). The entire assay and library construction can be carried out in a simple protocol involving Tn5 transposase insertion followed by PCR. Tn5 integrates its adaptor payload into regions of accessible chromatin, whereas steric hindrance in less accessible chromatin makes such transposition less probable. Therefore, amplifiable DNA fragments suitable for high-throughput sequencing are preferentially generated at locations of open chromatin. ATAC-seq has enabled the epigenomic characterization of latent HIV provirus indicating the presence of chromatin-based structural barriers to viral gene expression (119, 120). It was shown that intact HIV proviruses were enriched for non-genic chromosomal positions and more frequently integrated away from active transcriptional start sites and accessible chromatin regions (56). Additionally, reactivation studies showed that the activation of HIV transcription increased chromatin accessibility downstream of the HIV genome (138).

ATAC with select antigen profiling by sequencing (ASAP-Seq) combines single-cell ATAC-Seq with simultaneous measurement of cell surface and intracellular protein markers (139). A recent study has used ASAP-Seq to discover cell surface protein signatures that may identify or enrich infected cells in people with HIV (121). The methods used were able to successfully detect and individually characterize lymphoid as well as myeloid cells with proviral DNA in the blood from people with HIV on ART. This strategy has broad application for in-depth analysis of the HIV reservoir within different cell populations, therapeutic interventions, and HIV cure studies. However, the major limitations of ASAP Seq are the requirement for the provirus to be in accessible chromatin and the difficulty in assessing proviral intactness due to short-read sequencing and transposase-based methodology. Another single-cell-based method called the phenotypic and proviral sequencing (PheP-Seq) was designed to evaluate the phenotype of patient-derived HIV-infected cells, using a single-cell NGS assay that permits to jointly profile phenotypic markers and selected parts of the chromosomal DNA from single cells (122).

#### RNA sequencing

Bulk RNA-seq technologies have been widely used to study gene expression patterns at the population level in the past decade. The advent of single-cell RNA sequencing (scRNA-Seq) provides unprecedented opportunities for exploring gene expression profiles at the single-cell level. While the initial scRNA-Seq methods (140) produced NFL transcripts, later methods like Smart-seq, SUPeR-seq, and MATQ-seq were able to produce full-length sequencing data. More recent techniques prefer to only capture and sequence the 3′ end (Drop-seq, DroNC-seq, SPLiT-seq) or the 5′ end (STRT-seq) of the transcript. The various scRNA-seq methods and their applications in HIV research have been summarized in depth elsewhere (141, 142). scRNA-Seq has been extensively applied to study host responses and the heterogeneity of HIV infection (143). Innovative single-cell RNA sequencing pipelines can detect HIV and host transcripts simultaneously. The technology has been extensively used to characterize latent CD4+ T cells from the blood (54). Meanwhile, some studies have also targeted CD4+ T cells in the cerebrospinal fluid (CSF) (52) and mature monocyte subsets (110) of HIV-infected individuals on suppressive ART.

The cell-associated HIV RNA and DNA single-genome sequencing (CARD-SGS) was the first assay developed to investigate fractional proviral expression of HIV RNA and the levels of HIV RNA in single HIV-infected cells (108). This assay combined improved isolation of intracellular RNA from PBMCs with end-point dilution analysis of HIV RNA-expressing cells and can determine the fraction of all proviruses that express HIV RNA, the levels of HIV RNA expression from proviruses in single cells, and the fractional proviral expression and levels of HIV RNA in infected cell clones. Therefore, CARD-SGS has the potential to identify cellular and proviral sources of persistent and rebound viremia, as well as the impact of ART and experimental approaches to cure HIV infection on proviral expression at the single-cell level. While CARD-SGS has a relatively high sensitivity and specificity, it requires multiple amplification cycles and resolution steps, making it labor-intensive and low throughput.

Envelope detection by induced transcription-based sequencing (EDITS) is an NGS-based high-throughput assay that utilizes RT-nested PCR amplification of multiply spliced env RNA (109). To measure inducible cell-associated HIV-1 RNA, mRNA was isolated and cDNA corresponding to the multiply spliced env mRNA was amplified by nested PCR using a forward primer upstream of the major splice donor site combined with a reverse primer permitting identification of splice junctions spanning diverse regions of the genome. The samples treated under various conditions and from different patients were barcoded, pooled, and sequenced simultaneously. This approach is efficient with respect to time and sequencing costs and allows accurate comparisons since input cDNA levels are effectively normalized. The accuracy of the EDITS measurements was verified by using limiting dilution assays and there was reasonable agreement between the maximum likelihood estimates of the diluted samples and quantitation by EDITS. As measured by the EDITS assay, the total inducible RNA reservoir was found to be significantly smaller in women than in men (109).

More recently, another high-throughput method called the HIV SortSeq assay has been developed to identify rare HIV-infected CD4+ T cells from individuals on ART upon latency reversal *ex vivo* (113). HIV SortSeq is based on the classical SortSeq method that is based on fluorescence-activated cell sorting followed by sequencing (144). Transcriptome analysis by HIV SortSeq identified not only a transcriptome signature of HIV-infected cells upon latency reversal but also cellular factors that can serve as therapeutic targets in HIV eradication strategies. However, the study was limited to examination of HIV-infected CD4+ T cells harboring induced HIV RNA upon latency reversal, not in the quiescent latent state. Another limitation is the capture of only a subset of HIV-infected cells in which HIV proviruses are reactivated by a single round of PMA/ionomycin stimulation. It has been shown previously that some HIV proviruses can only be reactivated on multiple rounds of stimulation. Therefore, assays like HIV SortSeq and EDITS are likely to underestimate the total inducible HIV reservoir.

#### Multi-omics

High-throughput single-cell RNA sequencing has transformed the understanding of complex cell populations, but it does not provide phenotypic information such as cell-surface protein levels. Cellular indexing of transcriptomes and epitopes by sequencing (CITE-Seq) is a single-cell phenotyping method that uses DNA-barcoded antibodies to tag individual cells that are then processed for scRNA-Seq (145). CITE-seq can characterize even rare cell types according to cell surface proteins and is compatible with available high-throughput single-cell sequencing methods. The first study to detect HIV transcripts in the CSF T cells using scRNA-Seq used CITE-seq to provide information on the identity of CNS viral reservoirs in people with HIV on ART (52). CITE-seq is a scalable method that measures multiplexed intranuclear protein levels and the transcriptome in parallel across thousands of nuclei, enabling joint analysis of transcription factor levels and gene expression (146). DOGMA-Seq, an adaptation of CITE-Seq, allows combined measurement of chromatin accessibility, gene expression, and protein from single cells (139). It was shown using DOGMA-Seq that HIV-1 transcription correlated with HIV-1 accessibility and host chromatin accessibility (147). Other multi-omics approaches have identified CXCL13^high^ cells and a subpopulation of HIV-infected cells with low expression of interferon-stimulated genes, which can contribute to efficient viral spread *in vivo* (148).

Multi-omics studies have a great potential to explore the latent viral reservoir, novel biomarkers, extreme phenotypes, and new biological pathways. Cell hashing is a complementary approach to pioneering genetic multiplexing strategies, where cells are labeled with sample-specific hashtags for downstream de-multiplexing and multiplex detection (149). This approach has been used for expanded CRISPR-compatible cellular indexing of transcriptomes and epitopes by sequencing (ECCITE-Seq), which is capable of high-throughput characterization of at least five modalities of information from each single cell (150). This method was recently used to reveal HIV persistence in expanded cytotoxic T-cell clones (151). ECCITE-seq is designed to enable interrogation of single-cell transcriptomes together with surface protein markers in the context of CRISPR screens, but can easily be modified to combine immunophenotype, clonotype, and transcriptome information. More recent innovative methods like SEC-seq can combine single-cell transcriptomics and phenotyping with quantification of secreted IgG from the same single cells (152). Such techniques could be adapted to quantify the cytokines and/or viral product release, providing additional insight into the nature of the HIV-infected cells.

Given its high-throughput nature and potential to explore multiple modalities in single cells from precious clinical samples, the scope of multi-omics is expanding rapidly. Integrative multi-omics approaches combined with machine learning and artificial intelligence (AI) have enabled accurate identification, quantification, and characterization of rare cells in cancer and are valuable tools for precision medicine (153 – 155). Applications of AI and machine learning include exploring large data sets, finding new relevant patterns, predicting outcomes, and understanding associations of the complex molecular networks presented in multiple dimensions with unbiased analysis. These tools are becoming an integral part of translational research and if explored extensively in the field of HIV could be useful to detect and eliminate the low abundance tissue reservoirs.

### Challenges and future perspectives

The proviral landscape is extremely heterogeneous due to HIV inter- and intra-host diversity (114, 142, 156), internal deletions, and hypermutations. Even though 300/10^6^ resting CD4+ T cells contain HIV proviruses, only a fraction of these is replication-competent (157). However, even defective proviruses may produce HIV antigens and perturb adaptive immunity *in vivo*, potentially contributing to the persistent immune activation observed in patients on ART (60). Hypermutated proviruses and proviruses that have defects in splice sites can still be transcribed and the resulting RNAs can be translated (158). Cells carrying these defective proviruses can be recognized and targeted by HIV-specific cytotoxic T lymphocytes (68). On the other hand, intact proviruses that lack overt fatal defects such as large deletions and hypermutations may still have minor defects that affect fitness.

The advent of new genetic research technologies, in particular, next-generation full-length sequencing, has enabled a high-resolution analysis of the cells harboring genome-intact, replication-competent HIV (159) and can distinguish defective proviruses from intact ones. Sequencing methods allow direct *ex vivo* assessments of the cells from ART-treated persons living with HIV and characterization of rare infected cells, such as in the CNS compartment (52, 160). Recent studies have suggested a dominant influence of the proviral integration site and of proviral transcriptional activity on the persistence of intact HIV proviruses (56, 57, 116, 161). Intact proviruses integrated in heterochromatin locations and displaying signs of deep latency appeared to have a selection advantage and persisted long term, likely due to very low or absent proviral transcription and ensuing protection from antiviral immune recognition. In contrast, intact proviruses integrated in genic euchromatin positions appeared to be actively selected against, presumably due to the more notable production of viral transcripts that can be sensed by cell-intrinsic immune recognition pathways.

Although labor-intensive and costly, full-length proviral sequencing provides numerous advantages over other methods that probe sub-genomic regions. All sub-genomic amplification strategies introduce bias, resulting in sequence data that may not be representative of the actual population of HIV proviruses present in a given sample (162). Sequencing allows for detailed analysis of the proviral landscape, such as identification of the frequency of intact and defective proviral species, phylogenetic sequence analysis, and detection of clonal intact proviral sequences (76, 80, 126, 127). Moreover, it provides an assessment of persistent HIV diversity across different cell subsets and tissues, and with longitudinal sampling, could provide insight into the dynamics of reservoir frequency and diversity over time (107, 127). Long-read sequencing enables the sequencing of difficult or previously inaccessible regions of the genome, resulting in more complete genome assemblies. It can be used to sequence full-length transcripts, provide information about alternative splicing, and detect structural variants and DNA modifications at single-base resolution. While most of these methods provide important information about the nature of the reservoir, they do not necessarily predict the inducibility of the provirus or a quantitative measure of the size of the reservoir.

RNA-based assays are able to estimate the number of RNA copies per cell in addition to the frequency of RNA-expressing cells (163). RNA sequencing is a good way to measure inducible HIV but not necessarily intact HIV. Direct *in situ* hybridization-based assays like HIV-RNAscope and DNAscope have been used to provide a better understanding of the cellular distribution, phenotype, and transcriptional state of the tissue-associated HIV reservoir *in vivo*. Assays measuring HIV RNA and protein would be the next step to measure transcription as well as translation-competent reservoir. Methods like RNA expression and protein sequencing can simultaneously measure proteins and mRNAs in single cells (164) but have not yet been explored in the context of HIV. Therefore, continuous efforts are needed to develop new approaches that can accurately measure and characterize the true minimal HIV reservoir that must be targeted for future curative strategies.

It should be emphasized that a single approach cannot be universally applied (165). The choice of assay in a study depends largely on factors such as the mechanism expected for viral clearance, technologies available, sample size, and resource limitations. Most of the non-sequencing assays either overestimate (PCR-based assays) or underestimate (QVOA) the size of the reservoir. Additionally, these methods may also require amplification of the virus by external stimulation. Sequencing methods can overcome this caveat by providing important information about the nature of the reservoir as it is; however, the limit of detection and cost of these methods remain a constraint. Moreover, sequencing-based assays might not be well-suited for large clinical trials, especially where the aim is to quantify the reservoir rather than to characterize it, especially in the context of early treatment and interventions. The next generation of assays designed to measure the HIV reservoir should have a wide dynamic range, adaptability to cover different cell types, the capacity to distinguish intact proviruses, avoid the need for external stimulation, and should be scalable for large studies.
